# Ask us! Adjusting experience‐based codesign to be responsive to people with intellectual disabilities, serious mental illness or older persons receiving support with independent living

**DOI:** 10.1111/hex.13436

**Published:** 2022-02-18

**Authors:** Marjolijn Heerings, Hester van de Bovenkamp, Mieke Cardol, Roland Bal

**Affiliations:** ^1^ Erasmus School of Health Policy and Management Erasmus University Rotterdam Rotterdam The Netherlands; ^2^ Research Centre Innovations in Care Rotterdam University of Applied Sciences Rotterdam The Netherlands

**Keywords:** care relationship, experience‐based codesign, intellectual disability, older persons, reflexivity, serious mental illness, supported living

## Abstract

**Introduction:**

Experience‐based codesign (EBCD) is a valuable tool for participatory quality improvement. However, the EBCD process needs to be adjusted to make it suitable for long‐term care. The focus of the improvement process needs to shift to the care relationship, as this is an important part of the quality of care in these settings. Furthermore, the EBCD process needs to be made more accessible to vulnerable populations.

**Methods:**

Through a participatory research approach, EBCD was adjusted to long‐term care. The research was conducted in two care organisations: one supporting people with serious mental illness and intellectual disabilities in independent living and one providing homecare services for older persons.

**Results:**

The participatory research resulted in the development of ‘Ask us!’—a method for critical reflective codesign. The research furthermore provided valuable lessons for participatory projects with vulnerable clients. A common problem with participatory research in long‐term care is ensuring the involvement of clients and informal carers. We report on various strategies developed to include experiences of a diverse set of services users, such as combining interviews with participant observation, photo‐voice and involving experts‐by‐experiences as co‐ethnographers. In close collaboration with an inclusive theatre company, these experiences were translated into 42 short videos on complex situations in the care relationship from the perspective of clients, professionals or informal carers. These videos instigate critical reflection and accelerate the participatory quality improvement process. Moreover, practical tools were developed to overcome barriers regarding the involvement of people with disabilities. These include the use of photo‐elicitation to enable participation of clients with disabilities in heterogeneous group discussions and involving experts‐by‐experience as proxies to share experiences of clients for whom participation in the ‘Ask us’ method remains inaccessible.

**Conclusion:**

The result of a robust participatory process, ‘Ask us!’ is a promising method for participatory quality improvement in long‐term care. The research furthermore generated lessons for involving vulnerable populations in participatory research and codesign.

**Patient or Public Contribution:**

Clients were involved as informants, sharing their experiences with the care relationship in interviews, photovoice and observations. They were also involved as consultants, helping to analyse input for the film scripts during data validation sessions.

## 1 INTRODUCTION

Experience‐based codesign (EBCD) is a promising method for involving clients, professionals and family members in improving the quality of care (see Box 1 for an overview of the EBCD process).^1^, ^2^, ^3^, ^4^, ^5^ Applying this method in long‐term care settings, such as supported living for people with intellectual disabilities (IDs), serious mental illness (SMI) or older persons however raises several design challenges.

BOX 1. **EBCD**

**Figure 1:**
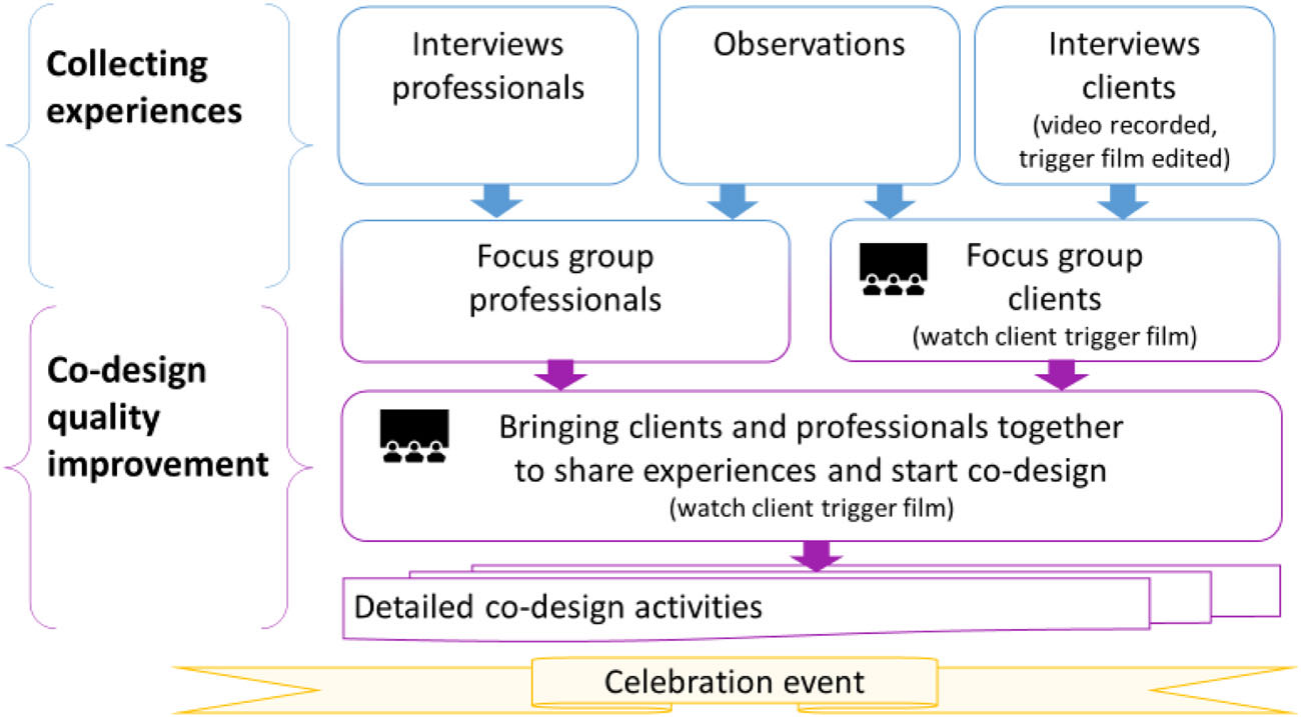
Experience‐based codesign process

EBCD (see Figure 1) is a process in which clients and professionals reflect on the quality of care and codesign improvements together. Informal carers are sometimes included in this process.^6^ The method consists of several phases. The first phase is to collect care experiences through interviews and observations. Of the interviews conducted with professionals, informal carers and clients, those with clients are videotaped and edited into a trigger film showing the various ‘touchpoints’ where clients experienced the service in a way that impacted them emotionally. The use of films is an important feature of EBCD, allowing emotional engagement in the codesign process. The trigger film also helps to create a level playing field in which client experiences receive enough attention and engage others towards change. Second, clients, professionals and sometimes informal carers reflect on their experiences in peer‐homogenous focus groups and identify areas for improvement. These group dialogues are facilitated through emotional mapping and, in the client group, by the client trigger film. Having peer‐homogenous groups gives the participants a safe space to share their experiences with peers and find common ground. Third, the separate groups come together to watch the client trigger film, deliberate the issues raised in their group dialogues and set common priorities for quality improvement. Smaller codesign groups representing all stakeholders are assembled for each priority and meet several times to develop organisational practices. The EBCD process concludes with a celebration that highlights the successes of the quality improvements.^1^, ^2^, ^3^

First, a shift in focus of the improvement process to the quality of the care relationship is needed. EBCD often focuses on specific aspects or moments in service delivery that impact the experiences of service users. However, in long‐term care, the care relationship is central to the quality of care.^7^, ^8^, ^9^, ^10^ The focus of EBCD in these settings should thus be on the care relationship. This care relationship is complex as it is often laden with value tensions.^11^, ^12^ Policy trends and care models, such as person‐centred care,^13^ recovery‐oriented care,^14^ active ageing^15^ and rehabilitation^16^ assign clients a more active role. In this context, values such as self‐determination, independence and community participation have become prominent. Putting those values into practice is complex as these values can be specified in different ways and may conflict with other values such as preventing harm. Self‐determination, for example, may mean respecting a client's decision to neglect standards of cleanliness or motivating them to clean their homes. Moreover, informal carers, clients and professionals can have different perspectives on these values, further complicating the care relationship.^12^, ^17^ To improve the quality of care in long‐term care, the EBCD process thus needs to shift focus to the ethical tensions in the care relationship.

Second, the EBCD process needs to be made more accessible to service users in long‐term care. Previous research has highlighted how the EBCD process can also produce vulnerabilities.^18^, ^19^ For instance, related to service users having to process rapid information flows or express themselves in heterogeneous group deliberations.^20^ Moreover, the length of the EBCD process also poses barriers to involvement as motivation declines or drop‐out occurs for other reasons.^4^ Previous adaptations of EBCD have accelerated the process by using existing films from a national archive to trigger responses in group sessions with clients, thus skipping the initial phase of interviews and observations. Using existing trigger films can have the advantage of making the process less threatening or challenging. A possible trade‐off could be staff engagement is aversively impacted. However, such an effect was not found in accelerated EBCD projects.^3^, ^4^, ^21^, ^22^ A key design challenge is therefore to better enable clients to participate in the dialogue sessions and to accelerate the process while fostering engagement. However, even when engaging in relational strategies and offering creative means of participation, such as photovoice, to include services users well beyond ‘the usual subjects’, certain groups of service users still often are excluded from participation. This results in specific experiences not being taken up in the codesign process.^19^, ^20^, ^23^ This raises the question how such otherwise excluded experiences can be included, in order to ensure a diverse range of experiences informs the quality improvement process.

In this paper, we report on a participatory project adjusting EBCD to long‐term care settings. This resulted in the method ‘Ask us!’. While a formal evaluation of ‘Ask Us!’ has not been conducted yet, the design process generated valuable lessons for participatory quality improvement with vulnerable populations, which are further explicated in this paper.

## 2 METHODS

### 2.1 Setting

We conducted participatory research in two care organisations situated in the same urban area in the Netherlands. The first was a community care organisation (CCO) providing supported housing (group homes and supported independent living) to people with IDs or SMI. The second provided homecare and other services to older persons (HO).

### 2.2 Project group for designing the instrument and developing the trigger films

We set up project groups for both the CCO and HO organisations to coproduce the research and the method for participatory quality improvement (see Table 1 for an overview). The size of the two project groups and their level of involvement differed, with fewer meetings in HO and less involvement of policymakers. This reflected differences in the two organisations, with HO having a much smaller policy/management layer. The number of meetings in HO could be reduced as the data collection and analysis was less complex, covering only one client group (older persons) instead of two (people with ID or SMI). Moreover, the participatory research in HO was conducted after the CCO project had finished and several decisions, for instance regarding the production of the videos, were already set. Members of HO were involved in the data analysis leading up to the content of the videos.

**Table 1:** Project groups

	Project group CCO	Project group HO
Participants	Two researchers; a member of a patient advocacy organisation; four policymakers; one expert‐by‐experience; a professional from each of the two collaborating teams	Two researchers; a member of a patient advocacy organisation; a policymaker; two community nurses and a manager (one community nurse and the manager were off the collaborating team)
Involvement	(1) Selection of teams; (2) data collection protocol including informed consent and topic lists; (3) data analysis and (4) designing the instrument	(1) Selection of teams; (2) data collection protocol including informed consent; (3) data analysis
Number of meetings	Six meetings: Five 90‐min meetings and one 4‐h workshop focusing on redesigning the group meetings and involving two additional experts on client participation	Two 90‐min meetings

Abbreviation: CCO, community care organisation; HO, homecare organisation.

### 2.3 Engaging service users, professionals and informal carers

We collaborated on developing one of the most important parts of the quality improvement method: the trigger films of both client, professional and informal carers experiences, with a care team for each client population (ID, SMI and older persons). Each team consisted of clients, professionals and informal carers, who acted as both informants and consultants. They provided input for the content of these films during in‐depth interviews about their experiences with the care relationship and informal conversations during participant observation and shadowing. CCO clients also participated using the photovoice method. As consultants, clients and professionals from each team participated in data validation sessions, helping to analyse the input for the film scripts (see Table 2 for an overview).

**Table 2:** Data collection

	Team CCO intellectual disabilities			Team CCO serious mental illness			Team HO older persons		
	Prof.	Cl.	Carers	Prof.	Cl.	Carers	Prof.	Cl.	Carers
Participant observation	12 Visits, 65 h total			12 Visits, 19 h total			–	–	–
Shadowing	–	–	–	–	‐	–	10 Visits, 60 h total		
Interviews	12	12	4	8	8	3	9	13	5
Photovoice	–	6	–	–	1	–	–	–	–
Interviews with peer‐support workers^a^	–	–	–	–	8	3	–	–	–
Group consultation on analysis (no. of participants)	9	5	–	8	5	–	7	5	–

Abbreviation: CCO, community care organisation; HO, homecare organisation.

^a^ Part of multiple teams catering for both intellectual disabilities and serious mental illness.

All three CCO and HO teams included professionals whose experiences were collected through interviews, participant observation (CCO) and shadowing (HO). Professionals were further involved through group discussions on the analysis of the interviews and observations serving as content for the trigger films. CCO professionals consisted of social care workers, while HO professionals included a community nurse, registered nurses, nurses in training and aides. The professionals of each team were asked to participate after introducing the research during a team meeting (CCO) or were asked by the nurse leading the team (HO). Reasons for declining included leaving the care team or being too busy.

Client recruitment proved more complex. Two things are specifically worth mentioning. First, it was difficult to recruit a diversity of clients with SMI because the affliction itself prevented them from participating. For example, clients refused interviews because they did not want to talk to ‘strangers’ or said their ‘voices’ did not allow it. Moreover, professionals who asked clients about being interviewed reported negative responses; in some cases, their already fragile care relationship was impacted. In response, we developed relational strategies to involve clients, for example by getting acquainted with them during coffee moments where they socialized.^20^, ^24^ The clients who did participate were not, however, a representative in terms of openness to contact. We, therefore, relied more on participant observation and informal conversation during these observations and developed further strategies to include the experiences of people unable to be interviewed. This included interviewing experts‐by‐experience about situations they had witnessed during their peer‐support work involving care relationships in the context of promoting self‐determination, independence and community participation.

For HO, we excluded people with dementia from interviews because they had difficulty recalling experiences with care professionals. We were, unfortunately, unable to include a sufficient diversity of clients from minority backgrounds, resulting in an underrepresentation of these groups. Partly these issues were resolved by including data from shadowing professionals.

It also proved difficult to interview informal carers in CCO. Service users acted as gatekeepers for contacting informal carers and their relationships were often complex.^20^ Moreover, of the limited number of informal carers that we were able to contact, several declined for various reasons. We resolved this problem by conducting additional interviews with family support workers, who elaborated on their own experiences with the services and that of other family members they supported. It was less relevant in HO to include informal carers because the older persons often managed their own care (see Table 2 for an overview).

### 2.4 Adjustments to the dialogue sessions

We organized a workshop to modify the method used to foster dialogue in both the peer‐homogenous groups and the joint EBCD event. The aim was to mitigate vulnerabilities by making the method more inclusive for clients who have difficulty processing information and speaking up in deliberative sessions and to shift the focus to the complex care relationship (see Table 1). The input for the workshop came from the researcher and the member of the patient advocacy organisation, who suggested ways of adapting the various EBCD phases. Their suggestions were based on interviews (*n* = 2) with EBCD project leaders involving people with SMI or ID, the literature on EBCD and practical experience with other client engagement methods. During the workshop, participants suggested and discussed these and other adaptations and modifications to the dialogue sessions until reaching a consensus.

## 3 RESULTS

**Figure 2:**
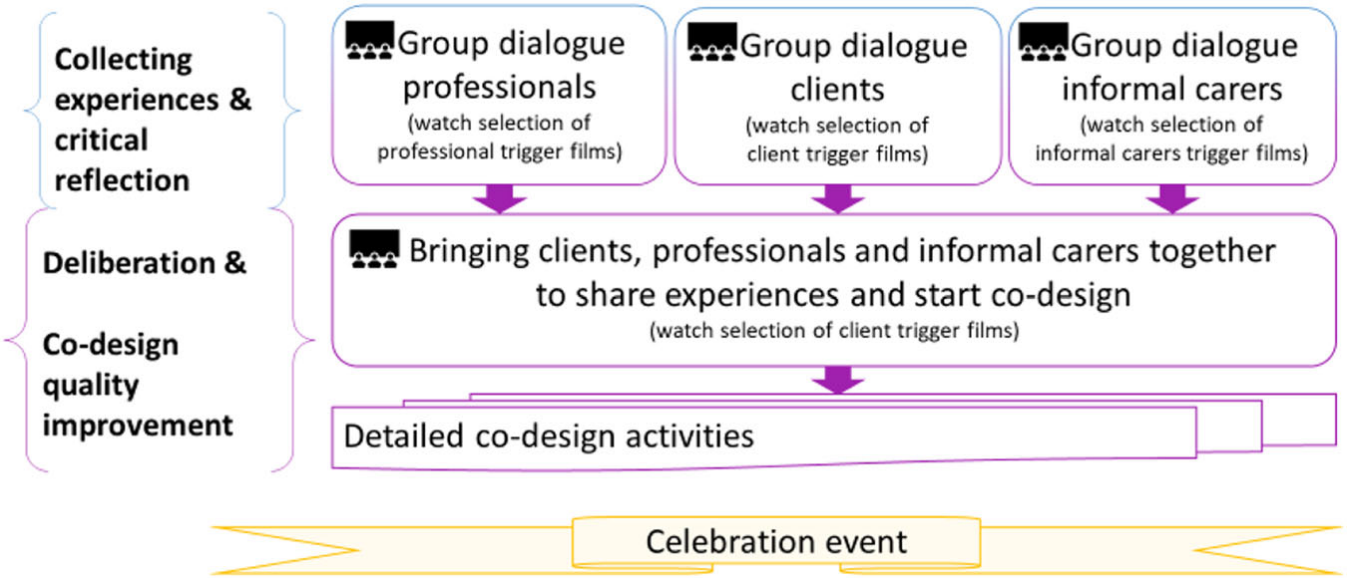
Ask us! redesigned experience‐based codesign

This participatory research resulted in the ‘Ask us!’ method. It consists of the 42 trigger films and a process for the various group and codesign sessions (see Figure 2). We first elaborate on the participatory process of developing the trigger films and reflect on lessons learned to include vulnerable groups in participatory research. We then describe the developed method for participatory codesign: ‘Ask us’ and explicate the lessons for participatory researchers aiming to involve vulnerable populations.

### 3.1 Trigger movies

The 42 trigger films are based on client, professional and informal carer experiences and are meant to accelerate the EBCD process and focus it on the complex long‐term care relationship. The films' length ranges between 1 and 3 minutes. The spoken language is Dutch; the films are made accessible to an international audience through English subtitling. For an overview we refer to Table S1, which lists the main themes for each film; for the full content, please visit www.eur.nl/en/eshpm/research/if-you-would-ask-us. The process we undertook to produce these trigger films differed from previous accelerated EBCD strategies.^21^, ^22^ Because many clients with SMI chose not to be interviewed on camera (similar to previous EBCD projects with this client group),^23^ we collaborated with theatre artists and developed scripts based on different data collection methods. As this also fitted our aim to accelerate the method, this format was used to produce films reflecting experiences of services users with SMI or ID, older persons and experiences of professionals and informal carers as well.

After discussing various possible formats, including documentary, digital storytelling and animation, the project group decided to give the films a realistic feel. The CCO members suggested an inclusive theatre company as a possible partner, as many of its actors had disabilities and received care from the CCO. They could contribute their own experiences to the creative process, making the films even more ‘real’. The theatre company's director suggested a *mis‐en‐scene* of close‐up monologues, to which the project team agreed. The format was therefore already set when we started the HO participatory process. The rest of the process ran similarly in CCO and HO.

Each film portrays complex situations in which values, such as self‐determination, an independent lifestyle or community participation conflict with other values, in which clients or informal carers may feel burdened by how care is enacted or organized, or in which clients, professionals or informal carers have different views on what constitutes good care. For example, one film considers the burden a client experiences when his personal care plan involves developing a personal hygiene routine and professionals are tasked with reminding him to shower on certain days. This practice leaves the client feeling misunderstood and restricted in his self‐determination. Another film, from a provider's perspective, shows how she struggles with clients who neglect their personal hygiene. The professional wants to intervene to foster the client's social acceptance and community participation but wonders whether doing so interferes with the client's self‐determination. Yet another film shows the perspective of the client's mother, who has been told by professionals that her son's personal hygiene choices are up to him, which she perceives as professional neglect. These examples not only show a complex situation from every perspective but also possible differences between clients, informal carers and professionals and, consequently, the need for deliberation, which is part of ‘Ask us!’.

The collaboration process involved translating the data of service users' experiences to the actual films in a series of steps.^25^ Throughout, we adhered to both a qualitative research logic and an artistic logic to ensure that the trigger films were both grounded in empirical research and able to engage audiences in reflection on their own experiences. Below, we describe the participatory process and translations steps leading up to the films.

The teams and the project group in each organisation were involved in developing content for the trigger films. We followed the logic of qualitative research by using ethnographic methods to collect data on the complex care relationship (see Table 2 for an overview). We used the same semi‐structured interviews for clients, professionals and informal carers, opening with the question ‘What is important to you in the care relationship you are involved in?’ Each named value prompted narrative exploration of situations in which this value was appropriately practised or proved difficult. When values such as self‐determination, an independent lifestyle or community participation were not named, the researcher provided prompts in the second half of the interview. Interviews were audio‐recorded and transcribed verbatim. The photovoice method included one session to explain how to operate the camera. Participants were then asked to take photos of what they did or did not like about where they lived. After 2 weeks, the photos were examined in interviews for themes related to the complex care relationship,^26^ with notes taken during the interviews being elaborated shortly after.

Different strategies were developed to include the experience of service users who chose not to partake in interviews or photovoice, or for whom these data collection methods were inaccessible.^27^ A first strategy included participant observation in the group homes part of CCO or shadowing of professionals in HO. This allowed us to observe the ‘enacted appreciations’ of clients, or their reactions to care practices without them having to vocalize these.^28^ Moreover, participant observation and shadowing allowed for many informal conversations with both clients and carers about care moments, shortly after they unfolded. These informal conversations were often much more accessible to clients compared to formal interviews and proved very valuable in collecting their experiences with care.

As a second strategy, we included the experiences of vulnerable clients in data collection by conducting interviews with experts‐by‐experience in their role as peer support workers. In the CCO organisation, experts‐by‐experience were part of care teams as peer support workers. As a result, they had witnessed many care moments and had many conversations with clients about their experiences with care. Similarly, the family‐experts‐by‐experience could share many experiences of carers, which complemented the interviews that were conducted. The interviews with these (family)‐experts‐by‐experiences followed the same format as the other interviews, although the focus was not on their own experiences but on those they had witnessed as part of their peer‐support work. These strategies allowed for the experiences of clients who chose not to partake in interviews or photovoice sessions to become part of the input for the trigger films. This is important as these clients seemed often more vulnerable and may have different experiences compared to clients who are willing and able to be interviewed.

To translate the different data of individual stories to common themes related to the complex care relationship, the first author conducted a thematic analysis. The analysis involved inductive coding of the data using Atlas‐ti software, identifying similarities and differences, and using axial coding to develop the themes. To refine this analysis, the researcher edited quotes per subtheme into comprehensible narratives and discussed them in various sessions with the project group and with clients, professionals and family support workers (see Tables 1 and 2). The narratives were then adjusted based on these discussions.

**Figure 3:**
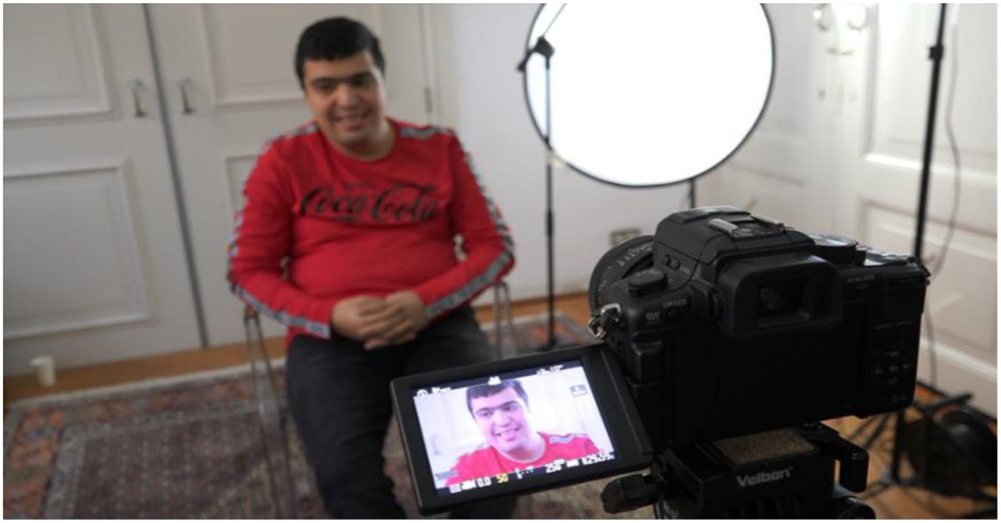
Filming

The narratives developed in consultation with the project groups and the teams were shared with the theatre company, to serve as the text for the filmed monologues. The director found the narratives too lengthy and in lack of poetic use of language that would feel like ‘normal talk’ while moving and engaging audiences at the same time. A playwright was engaged to produce another translation based on artistic logic. He rewrote the narratives as monologues, changing most of the original phrasing. To prevent a loss of thematic content, the researcher had two sessions with the playwright to revise the text. Between these two sessions, the researcher also discussed the monologues with one of the CCO experts‐by‐experience (and theatre maker) and took her suggestions on board in the second meeting with the playwright. The project group checked and approved the final versions of the monologues, which the actors then rehearsed and recorded on camera. The rehearsal of the monologues by the actors proved a further check on the integrity of the monologues as actors were encouraged to use their own experiences as part of their interpretation of the monologues. Some of the actors had experience as clients of CCO or of a similar organisation for supported independent living. Most of the other actors had experiences similar to those of professionals, as the theatre company also served as community day‐care, and the formal role of these actors was that of support staff in this setting. The interpretation of the actors also added a final layer of translation following an artistic logic, as the performances were based on different intentions (or emotions) to create a diverse pallet of films and to move their audience (see Figure 3 for an image of the process).

### 3.2 ‘Ask Us!’ dialogue sessions

We now turn to the details of the different dialogue session part of the participatory quality improvement method ‘Ask us!’ and explicate the lessons learned, which are of interest to a wider audience of participatory researchers aiming to involve vulnerable populations.

The first phase of the method focuses on collecting care relationship experiences and reflecting critically on the tensions between values associated with clients' playing an active role. In this phase, clients, professionals and informal carers engage in separate group dialogues in which they share experiences and reflect critically after viewing selected trigger films specific to each peer‐homogenous group. The main issues for making the EBCD more accessible to vulnerable populations were first to keep the input manageable and prevent information overload; second, to use visuals over textual information.

For the purpose of managing information overload, we reduced the time of the trigger film to a selection of 3–5 films, lasting no more than ten minutes rather than the usual 30‐min trigger film in EBCD. The selection is made in consultation with an expert‐by‐experience and professional familiar with the team engaged in the 'Ask Us!' method.

**Figure 4:**
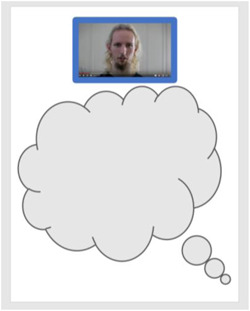
Worksheet after watching trigger films

After watching the selected videos, the participants reflect on their own experiences triggered by the films. We modified the EBCD emotional mapping method to make it more accessible to a diverse group of clients and to focus on the complex care relationship. For this, we developed various visual materials. Clients are asked whether watching the film evoked a recent experience in their care that still moves them emotionally or that they think about at times. They are asked to choose a worksheet belonging to one of the films that triggered this memory (see Figure 4), or to choose a blank worksheet if this fits best, and to affix emoticon stickers to it or add drawings or words reflecting their experience. Each worksheet is then matched with the corresponding visual representation of the film and assigned the same colour code on the wall. Group facilitators initiate group dialogue by pointing out emotional responses on the wall and inviting people to share their stories, highlighting aspects of the complex care relationship. During these critical reflections, facilitators work towards common themes, which were made visual on the sheets by drawing or using preprinted pictograms.

Next, the participants prioritize themes for quality improvement by placing three stickers with one or more of the themes. This way a single theme is selected for deliberation at the joint event. Reducing the number of themes compared to the original EBCD setup not only makes the joint event more manageable but also promotes the in‐depth deliberation that is at the core of this instrument and required in complex situations in which clients, professionals and informal carers may have differing perspectives.

Another key aspect of participatory research with vulnerable populations, which emerged during the process of redesigning the various dialogue sessions, is making sure their inputs are responded to in ways that encourage further participation even when their input is not further incorporated within the participtory quality improvement process or reasearch. This requires designing additional processes in which these concerns can be addressed. In EBCD and in 'Ask Us!', this for instance is important in the process of selecting themes for quality improvement. This is a delicate process as it can be overwhelming for some clients to voice a concern or area for improvement in the peer‐homogenous group and they may feel hurt or demotivated if their concern is not selected. An additional procedure was therefore designed in which experts‐by‐experience coach clients to address these concerns in an appropriate setting, for instance in their individual care relationship, in the client council or in a team meeting of professionals. This procedure is an addition to the more generic support available for clients before, during and after the sessions.^23^, ^29^

An important aspect of the design of participatory research is to reduce power imbalances to better enable clients to contribute to group discussions in heterogeneous settings. This requires the development of additional tools to enable them to take a stand and to shift some of the responsibility for making their voices heard to other participants. For adjusting EBCD, this is especially relevant in the second EBCD phase, where clients, professionals and informal carers deliberate on the themes chosen by each peer‐homogeneous group. One of the tools to foster a level playing field are the films shot from the clients' perspective on each theme watched at the beginning of this session. Smaller mixed subgroups then deliberate similarities and differences between the different groups' perspectives on each of the three themes. Participating in the deliberations with professionals and informal carers can be particularly challenging for clients. To address this, we developed a photo‐elicitation method. Various cards with photographs relevant to the themes are laid on the table. Clients can take a card and hold it up to signal if they wish to contribute to the conversation. This shifts the responsibility to the other participants to ask clients what they wish to contribute without clients having to verbalize this mentally before taking the floor. Between the deliberations on each theme, each group shares its main points with the whole group and the facilitator uses this information to construct a theme for quality improvement. At the end of the event, mixed groups are formed to codesign improvements for each theme. These smaller codesign sessions follow the blueprint developed by MH‐ECO.^5^ During these co‐design sessions, facilitators can introduce already invented best practices so not to reinvent the wheel. In those cases the co‐design process consists of adjusting these best practices to the specific needs and context of the clients, professionals and informal carers. A key point is these best practices should be offered to inspire participants, while making sure participants remain ownership over the co‐design process.

## 4 DISCUSSION

In this participatory research project, clients, professionals, informal carers, experts‐by‐experience, family support workers, researchers and policymakers collaborated on developing a method for participatory quality improvement of the complex care relationship in long‐term care focusing on self‐determination, an independent lifestyle and community participation.

EBCD was a valuable source because it involves a process whereby client, professional and informal carer engage in reflection, deliberation and codesign, but it needed to be redesigned for long‐term care by:

1. Accelerating the process and focusing on the complex care relationship: 42 short trigger films were developed addressing the dilemmas and burdens experienced by clients, informal carers and professionals in the care relationship in supported housing for people with IDs or SMI and homecare services for older persons.
2. Reducing existing and preventing new vulnerabilities: the various group dialogues central to EBCD were modified to make the method more accessible for clients with SMI or ID or for older persons.

The resulting method, ‘Ask us!’, brings together critical reflection, deliberation and codesign in a comprehensive process that has the potential to improve services.

‘Ask us!’ allows organisations to involve professionals in critical reflection, helping them to better handle complex situations, and to engage clients, professionals and informal carers in redesigning their services so as to empower service users. It should be noted, however, there has yet to be a formal evaluation, which will be an important next step in the refinement of this method for participatory quality improvement. Collaborating care organisations and audiences at presentations and film viewings—including client representatives and professionals—however, have responded positively to the method. Both clients and professionals expressed that the content of the videos was recognisable to them and the videos could be useful in fostering conversation on quality of care.^30^

The participatory process through which this method was developed yields several lessons to researchers aiming to involve vulnerable populations in participatory research. Previous research on involving vulnerable populations has highlighted how ensuring participation requires creating relationships with service users before engagement, including creative methods, to enable them to share experiences and involving service users at their own pace.^19^, ^20^, ^23^, ^31^ These strategies however proved insufficient in our research to ensure diversity in the experiences included. While it remains preferable both from a methodological and ethical standpoint to ensure direct involvement of vulnerable groups, alternative strategies might be needed. When methods of direct involvement such as interviews, focus groups or creative methods like photovoice prove inaccessible to certain clients or family members, their important experiences are excluded from the research or quality improvement process. To overcome this barrier, such methods of direct involvement can be supplemented with other methods, such as observations of enacted appreciations^28^; informal conversations during participant observations and involvement of (family) experts‐by‐experience as co‐ethnographers.

Another important lesson is to develop a process through which the input of clients that is not part of the further codesign process is still taken up and responded to within the care organisation. This is an important addition to current research on involving vulnerable populations in codesign processes in which the need of having counselling available during and after sessions is often highlighted.^23^, ^31^ While this is an important part of caring for participants' well‐being, responding to their concerns regarding the quality of care that were not selected in the design process is also needed.

Lastly, the traditional EBCD process already uses creative means to lift some of the power imbalances in mixed‐group sessions: the trigger films showing clients' experiences. We added a photo‐elicitation method to further enable clients to take a stance and voice their concerns and professionals and informal carers to hear these.

These lessons emerged in our process of redesigning EBCD to fit long‐term care settings. They are valuable as well to other researchers in involving vulnerable populations in participatory research or codesign projects.

## CONFLICT OF INTERESTS

The authors have no conflict of interests to declare.

## AUTHOR CONTRIBUTIONS

Marjolijn Heerings: Conceptualisation; investigation; methodology; project administration; writing the original draft. Hester van de Bovenkamp: Conceptualisation; funding acquisition; methodology; project administration; supervision; writing‐review & editing. Mieke Cardol: Conceptualisation; methodology; writing‐review & editing. Roland Bal: Conceptualisation; funding acquisition; supervision; writing‐review & editing.

## ETHICS STATEMENT

The ethical board of Erasmus Medical Centre judged the study as not requiring ethical approval under Dutch law (MEC‐2017‐122).

## Supporting information

Supporting information.

## Data Availability

The data supporting the findings of this study are available from the corresponding author upon reasonable request.

## References

[1] Bate P , Robert G . Experience‐based design: from redesigning the system around the patient to co‐designing services with the patient. Qual Saf Health Care. 2006;15(5):307‐310. 17074863 10.1136/qshc.2005.016527 PMC2565809

[2] Bate P , Robert G . Bringing User Experience to Healthcare Improvement: the Concepts, Methods and Practices of Experience‐Based Design. Oxford; 2007.

[3] Robert G . Participatory action research: using experience‐based co‐design to improve the quality of healthcare services. In: Coulter A , Calabrese JD , Locock L , Ziebland S , eds. Understanding and Using Health Experiences: Improving Patient Care. Oxford University Press; 2013.

[4] Donetto S , Tsianakas V , Robert G . Using Experience‐Based Co‐design (EBCD) to Improve the Quality of Healthcare: Mapping Where We are Now and Establishing Future Directions. King's College London; 2014.

[5] Palmer VJ , Weavell W , Callander R , et al. The Participatory Zeitgeist: an explanatory theoretical model of change in an era of coproduction and codesign in healthcare improvement. Med Humanit. 2019;45(3):247‐257. 29954854 10.1136/medhum-2017-011398 PMC6818522

[6] Hackett CL , Mulvale G , Miatello A . Co‐designing for quality: creating a user‐driven tool to improve quality in youth mental health services. Health Expect. 2018;21(6):1013‐1023. 29707865 10.1111/hex.12694 PMC6250867

[7] de São José JMS . Care and the shadow of the fourth age: how does home care get caught up in it and how does it stay away from it? Ageing Soc. 2020;40(3):643‐662.

[8] Lindvig GR , Larsen IB , Topor A , Bøe TD . ‘It's not just a lot of words’. A qualitative exploration of residents' descriptions of helpful relationships in supportive housing. Eur J Soc Work. Published online October 24, 2019. 1‐13. https://www.tandfonline.com/doi/full/10.1080/13691457.2019.1682523

[9] Strandås M , Bondas T . The nurse–patient relationship as a story of health enhancement in community care: a meta‐ethnography. J Adv Nurs. 2018;74(1):11‐22. 28702952 10.1111/jan.13389

[10] Topor A , Ljungberg A . “Everything is so relaxed and personal”—The construction of helpful relationships in individual placement and support. Am J Psychiatr Rehabil. 2016;19(4):275‐293.

[11] Heerings M , van de Bovenkamp H , Cardol M , Bal R . Tinkering as collective practice: a qualitative study on handling ethical tensions in supporting people with intellectual or psychiatric disabilities. Ethics Soc Welf. 2021;57:1‐18.

[12] Heerings M , van de Bovenkamp H , Cardol M , Bal R . Ethical dilemmas of participation of service users with serious mental illness: a thematic synthesis. Issues Ment Health Nurs. 2020;41(4):283‐295. 31990626 10.1080/01612840.2019.1667459

[13] Claes C , Van Hove G , Vandevelde S , van Loon J , Schalock RL . Person‐centered planning: analysis of research and effectiveness. Intellect Dev Disabil. 2010;48(6):432‐453. 21166549 10.1352/1934-9556-48.6.432

[14] Farone DW . Schizophrenia, community integration, and recovery implications for social work practice. Soc Work in Ment Health. 2006;4(4):21‐36.

[15] Whitehead PJ , Worthington EJ , Parry RH , Walker MF , Drummond AE . Interventions to reduce dependency in personal activities of daily living in community dwelling adults who use homecare services: a systematic review. Clin Rehabil. 2015;29(11):1064‐1076. 25587088 10.1177/0269215514564894 PMC4607918

[16] Farkas M , Anthony WA . Psychiatric rehabilitation interventions: a review. Int Rev Psychiatry. 2010;22(2):114‐129. 20504052 10.3109/09540261003730372

[17] Pols J , Althoff B , Bransen E . The limits of autonomy: ideals in care for people with learning disabilities. Med Anthropol. 2017;36(8):772‐785. 28836876 10.1080/01459740.2017.1367776

[18] Dimopoulos‐Bick T , Dawda P , Maher L , Verma R , Palmer V . Experience‐based co‐design: tackling common challenges. J Health Des. 2018;3(1):86‐93.

[19] Moll S , Wyndham‐West M , Mulvale G , et al. Are you really doing ‘codesign’? Critical reflections when working with vulnerable populations. BMJ Open. 2020;10(11):e038339. 10.1136/bmjopen-2020-038339 PMC7640510 33148733

[20] Mulvale G , Moll S , Miatello A , et al. Codesigning health and other public services with vulnerable and disadvantaged populations: insights from an international collaboration. Health Expect. 2019;22(3):284‐297. 30604580 10.1111/hex.12864 PMC6543156

[21] Locock L , Robert G , Boaz A , et al. Testing accelerated experience‐based co‐design: a qualitative study of using a national archive of patient experience narrative interviews to promote rapid patient‐centred service improvement. Health Serv Deliv Res. 2014;2(4):1‐122. 25642558

[22] Locock L , Robert G , Boaz A , et al. Using a national archive of patient experience narratives to promote local patient‐centered quality improvement: an ethnographic process evaluation of ‘accelerated’ experience‐based co‐design. J Health Serv Res Policy. 2014;19(4):200‐207. 24840387 10.1177/1355819614531565

[23] Larkin M , Boden ZV , Newton E . On the brink of genuinely collaborative care: experience‐based co‐design in mental health. Qual Health Res. 2015;25(11):1463‐1476. 25829467 10.1177/1049732315576494

[24] Springham N , Robert G . Experience based co‐design reduces formal complaints on an acute mental health ward. BMJ Open Qual. 2015;4(1):u209153.w3970. 10.1136/bmjquality.u209153.w3970 PMC4693090 26734433

[25] Guggenheim M . The media of sociology: tight or loose translations? The media of sociology. Br J Sociol. 2015;66(2):345‐372. 26072682 10.1111/1468-4446.12125

[26] Drew G . From photographs to findings: visual meaning‐making and interpretive engagement in the analysis of participant‐generated images. Vis Stud. 2014;29(1):54‐67.

[27] Drew S , Guillemin M . From photographs to findings: visual meaning‐making and interpretive engagement in the analysis of participant‐generated images. Vis Stud. 2014;29(1):54‐67.

[28] Pols J . Enacting appreciations: beyond the patient perspective. Health Care Anal. 2005;13(3):203‐221. 16223211 10.1007/s10728-005-6448-6

[29] Cooper K , Gillmore C , Hogg L . Experience‐based co‐design in an adult psychological therapies service. J Ment Health. 2016;25(1):36‐40. 26670265 10.3109/09638237.2015.1101423

[30] Wiesman A . Balancing between self‐reliance and frailty in long‐term care. Filmed monologues initiate a conversation about the care relationship. In: ZonMw , ed. Interview series Care Relationship. ZonMw; 2021.

[31] Mulvale A , Miatello A , Hackett C , Mulvale G . Applying experience‐based co‐design with vulnerable populations: lessons from a systematic review of methods to involve patients, families and service providers in child and youth mental health service improvement. Patient Exp J. 2016;3(1):117‐129.

