# Supplementary material for: Ask us! Adjusting experience‐based codesign to be responsive to people with intellectual disabilities, serious mental illness or older persons receiving support with independent living
Source: Health Expect. 2022 Feb 18;25(5):2246–54. doi: 10.1111/hex.13436 (PMC9615044; doi:10.1111/hex.13436)
Supplement: Supplementary file 1 — Supporting information. [file HEX-25--s001.docx]

Table 1 Overview of the themes for each film

|  | **Clients** | **Professional** | **Informal carers** |
| --- | --- | --- | --- |
| Supported independent living for people with serious mental illness or intellectual disability | Develop a close relationship with professionals; provide support in a way that contributes to empowerment. | Build a close relationship while maintaining professional distance; use of social media; availability outside official hours. | Professionals invest in getting to know the client well; take the client seriously, engage in an equal relationship and have continuity in staffing. |
|  | Not be stigmatized by a psychiatric diagnosis and be supported in limiting self-stigma; have professionals use their knowledge of mental health to deepen support; foster dialogues about the content of hallucinations, delusions, and about suicidal ideation. | Get to know the client beyond the disability-related identity; use professional knowledge to deepen contact. |  |
|  | Not let goals in personalized care plan limit choices in daily life, while also motivating clients to achieve these goals. Support personal goals even when these do not seem to contribute to developing an independent lifestyle or community participation. | Motivate clients to develop an independent lifestyle or community participation, or support clients in their self-determined goals even when these do not contribute to becoming independent. | Professionals motivate clients to work on goals that are important to clients or are important for developing a more independent lifestyle or community participation. |
|  | Allow room to make mistakes (dignity of risk) while also relationally engaging in choice-making and sometimes limiting choices to support second-order desires. | Let client experience the consequences of self-determined actions as part of their learning process, or prevent harm. | Clients sometimes present themselves as more independent than they are, professionals should be wary of overestimating clients and make sure they provide sufficient support to prevent harm. |
|  | Pay attention to (minor) strengths and progress and let clients do certain things themselves while also providing support where needed. | Intervene when clients do not keep up standards of (personal) hygiene to prevent stigmatization and promote social inclusion or accept this as personal preference/choice. |  |
|  | Engage in dialogue and provide support on ‘delicate’ subjects such as spirituality; existential questions and need for intimacy & sexuality. | Support clients in finding ways to meet their need for intimacy & sexuality or prevent abuse by others. | Difficulties when professionals feel client desires intimacy/sexuality while family members feel this is not the case. |
|  | Not place taboos on wanting to stop taking psychotropic medications; know preferences on how to be approached in times of crisis. | How to deal with clients who are in crisis but refuse support. |  |
|  | Transition to living independently and facing stigma and loneliness. | Promote independent living or prevent loneliness and other risks; abuse and clients engaging in criminal activities. | Professionals should encourage clients to participate in the community to alleviate loneliness while also preventing risk of abuse. |
|  | Acknowledge difficulties of moving to a new home/neighbourhood and provide support in community participation. | Stimulate clients to take part in community activities or organize social meetings for clients to get together. |  |
|  | Involve family members and support the improvement of relationships with family members while also guarding against overburdening family members with the role of professional carer; limit family members’ over-involvement and other negative influences on recovery or development. | Collaborate with family members on supporting client; prevent over-involvement of family members and deal with family members who fear negligence while the professionals’ aim is to support self-determination and an independent lifestyle. | Professionals provide information about how support is provided; professionals provide information on the client; use knowledge of family members to get to know clients; negligence by professionals leads to extra burden for informal carers; family members want to support client as family members, not as informal carers. |
| Home care services for older adults | Abide by preferences regarding care scheduling and whether care is provided by a man or a woman. | Not always being able to abide by preferences due to case load or other organizational complications. | Abide by the client’s preferences; continuity in care. Inform informal carers on delivery of care. |
|  | Abide by preferences regarding the client’s home; smoking, pets, professionals taking off their coat; professionals ringing the doorbell instead of using a key. |  |  |
|  | Abide by preferences regarding how care is performed or when allocated care can be skipped on a given day. | Abide by client’s preference not to receive scheduled care or motivate clients to undergo care to prevent harm (e.g. showering; stockings etc.). | Clients should be motivated to accept the allocated care they need to prevent harm. |
|  | Don’t rush, so clients can take the time they need to do things independently (e.g. dressing themselves after showering). | Support independence by not doing extra non-allocated work. Motivate clients to accept support so they have enough energy for other things important for their quality of life. | Negligence due to professionals leaving too much for clients to do by themselves and having no time for social contact. |
|  | Support clients in (minor) needs even when these are not part of allocated care (including social contact). | Take extra time for social contact and be sensitive to clients’ needs even when not part of allocated care or stick to allocated care and leave other tasks for older adults or their family members to do to foster active ageing. | Support needed for informal carer as well. |
